# Predictive factors of adherence to frequency and duration components in home exercise programs for neck and low back pain: an observational study

**DOI:** 10.1186/1471-2474-10-155

**Published:** 2009-12-09

**Authors:** Francesc Medina-Mirapeix, Pilar Escolar-Reina, Juan J Gascón-Cánovas, Joaquina Montilla-Herrador, Francisco J Jimeno-Serrano, Sean M Collins

**Affiliations:** 1 Department of Physical Therapy, University of Murcia, Murcia, Spain; 2 Department of Public Health and Preventive Medicine, University of Murcia, Murcia, Spain; 3 Department of Physical Therapy, University of Massachusetts, Lowell, MA, USA

## Abstract

**Background:**

Evidence suggests that to facilitate physical activity sedentary people may adhere to one component of exercise prescriptions (intensity, duration or frequency) without adhering to other components. Some experts have provided evidence for determinants of adherence to different components among healthy people. However, our understanding remains scarce in this area for patients with neck or low back pain. The aims of this study are to determine whether patients with neck or low back pain have different rates of adherence to exercise components of frequency per week and duration per session when prescribed with a home exercise program, and to identify if adherence to both exercise components have distinct predictive factors.

**Methods:**

A cohort of one hundred eighty-four patients with chronic neck or low back pain who attended physiotherapy in eight primary care centers were studied prospectively one month after intervention. The study had three measurement periods: at baseline (measuring characteristics of patients and pain), at the end of physiotherapy intervention (measuring characteristics of the home exercise program) and a month later (measuring professional behaviors during clinical encounters, environmental factors and self-efficacy, and adherence behavior).

**Results:**

Adherence to duration per session (70.9% ± 7.1) was more probable than adherence to frequency per week (60.7% ± 7.0). Self-efficacy was a relevant factor for both exercise components (p < 0.05). The total number of exercises prescribed was predictive of frequency adherence (p < 0.05). Professional behaviors have a distinct influence on exercise components. Frequency adherence is more probable if patients received clarification of their doubts (adjusted OR: 4.1; p < 0.05), and duration adherence is more probable if they are supervised during the learning of exercises (adjusted OR: 3.3; p < 0.05).

**Conclusion:**

We have shown in a clinic-based study that adherence to exercise prescription frequency and duration components have distinct levels and predictive factors. We recommend additional study, and advise that differential attention be given in clinical practice to each exercise component for improving adherence.

## Background

Exercise therapy is often indicated in the treatment of neck and low back pain [1,2]. Exercises are individually taught and prescribed for home [3]. They can vary greatly in content and method of delivery [3,4]. Home exercise programs (HEPs) often include components of intensity, duration and frequency.

Several systematic reviews provide evidence of the benefits of exercise among people with chronic neck and back pain [1,2]. However, several studies report that adherence to exercise is often a serious problem for these individuals [5-10]. Depending on differences in the definition utilized for adherence and its measurement, estimates of how many patients complete their exercises according to prescriptions vary, but converge on a figure of 50% or less [5-10].

Many factors have been identified that affect HEP adherence. These include characteristics of patients such as socio-demographic variables, adherence history, motivation and social support, their illness and environment, aspects of the prescribed program and of the provider [11]. Some factors, such as perceived barriers associated with daily routine or self-efficacy, have been consistent predictors of adherence across studies [5,8,12,13]. Other factors, such as pain intensity, are variably implicated as a predictor of adherence [5,8,10].

There is evidence that in healthy people the determinants of adherence to frequency and duration components of HEP are different [14]. However, it remains unclear whether those differences apply to patients receiving physiotherapy and HEPs for chronic neck and low back pain. Our understanding remains scarce in this area on both, levels of adherence and determinants of adherence for distinct components of exercise. Part of the problem may be that exercise has not often been thought of a multidimensional behavior in adherence studies for HEPs. These studies usually measure only selected components, such as frequency [5], or combine the components into one measure of exercise [7]. Therefore, research has not addressed different determinants of adherence to these HEP components. However, documenting predictive factors of adherence to HEP components may contribute to the development of more effective interventions to improve adherence [14].

The aims of this study are to determine whether patients with neck or low back pain have different rates of adherence to exercise components of frequency per week and duration per session when prescribed with a home exercise program, and to identify if adherence to both exercise components are predicted by distinct factors.

## Methods

### Design

A cohort of subjects with chronic non-specific neck or low back pain were studied prospectively during one month after intervention to determine their short-term adherence to a home exercise prescription provided by their physiotherapist. Aims of intervention were to provide modalities of physical therapy and give patient education for HEP and others self-management strategies.

### Participants

The study was approved by the Bioethic Committee of University of Murcia, Spain. During a six month period all subjects with chronic (more than 3 months) non-specific neck or low back pain receiving physiotherapy intervention in eight primary care centers of the Health Service of Murcia (Spain) were recruited consecutively into the study. Exclusion criteria were: younger than 18 or older than 70 years, unable to read or write, cognitive deficit (e.g. Alzheimer disease or senile dementia), unable to attend all sessions of physiotherapy, and stop HEP by physiotherapist's prescription. Patients provided written informed consent prior to participation.

### Procedures of data collection

At the baseline (before physiotherapy intervention) we collected, from participants self report and by a structured interview, information on patients and pain characteristics. Subjects were blinded to the research questions.

Over next four weeks, patients attended at health center for physiotherapy treatment, education and follow-up of HEP. On the last day of physiotherapy intervention (four weeks following baseline), the physiotherapist (using a registration form that was designed "ad hoc") provided data from records on the characteristics of the HEP that patients should do during next month. Physiotherapists were not blinded to research questions. One month later subjects were asked by a postal questionnaire about the quality of clinical encounters with the physiotherapist during the intervention program, environmental factors and adherence behavior to home exercise during the last week. Pharmacological treatments were not collected.

### Measurement of predictive factors

Table 1 shows the factors that were assessed before, during and after physiotherapy treatment.

**Table 1:** Study measures

Measurements	Baseline interview (before intervention)	Registration form (on last day of intervention)	Post-intervention questionnaire (one month later)
**CHARACTERISTICS OF PATIENT**			
Gender	X		
Age	X		
Education level	X		
Work participation	X		
Sick leave	X		
Use of physiotherapist in previous episodes of pain	X		
Participation in home program in previous episodes	X		
			
CHARACTERISTICS OF PAIN			
Location	X		
Pain intensity	X		
Disability related to pain	X		
			
CHARACTERISTICS OF HOME EXERCISE PROGRAM			
Home exercise program		X	
Type of exercises		X	
Total number of exercises		X	
Days at week		X	
Duration of each session		X	
			
ENVIRONMENT FACTORS AND SELF-EFFICACY			
***Perceived barriers and social support***			
Having too little time			X
Exercises do not fit daily routine			X
Emotional support			X
***Self-efficacy***			
Self-efficacy			X
			
PHYSIOTHERAPISTS' BEHAVIOURS IN CLINICAL ENCOUNTERS			
***General information***			
Clarifying doubts and questions from patients			X
Giving information about illness			X
Justifying usefulness of advises			X
***Instructions for carrying out the exercises***			
Giving written instructions			X
***Follow-up of exercises***			
Supervising exercises in health care centre			X
Asking about adherence at home			X
***Patient's assesment***			
Satisfaction			X
			
ADHERENCE BEHAVIOUR			
Component of frequency per week			X
Component of duration per session			X

#### Characteristics of patients

Subjects reported their age (years), gender (male/female), education level (without studies/primary/secondary/university), work participation (yes/no), sick leave (yes/no) and use of physiotherapy and home exercise in previous episodes of pain (yes/no). Following similar criteria to previous study [5], age was stratified into three age ranges, as shown in Table 2.

**Table 2:** Baseline information (% or mean) of respondents and non-respondents.

Variables	Groups		Difference between groups
	
	Respondents % or mean (n_1 _= 184)	Non-respondents % or mean (n_2 _= 83)	Respondents minus Non-respondents % (95%CI)
**CHARACTERISTICS OF PATIENTS**			
Gender			
Male	19.6	9.9	9.7 (-0.5 to 19.6)
Age			
18-39	30.4	48.7	-18.3 (-31.2 to -5.3)
40-59	46.2	44.9	1.3 (-11.8 to 14.5)
>59	23.4	6.4	17.0 (8.8 to 25.1)
Education level			
Without studies	20.3	14.5	5.8 (-4.0 to 15.8)
Primary studies	51.7	63.2	-11.5 (-24.5 to 1.8)
Secondary and University	27.9	22.4	5.5 (-5.9 to 17.1)
Work participation			
Yes	60.2	66.2	-6.0 (-19.0 to 7.0)
Sick leave			
Yes	40.0	37.0	3.0 (-13.9 to 19.9)
CHARACTERISTICS OF PAIN			
Location			
Neck pain	63.1	66.2	-3.1 (-15.9 to 9.6)
Pain-disability			
High level	61.8	65.8	-4.0 (-16.8 to 8.9)
			
Pain intensity (0-10), mean (SD)	7.2 (1.8)	7.2 (2.2)	0.0 (-0.5 to 0.5)

#### Pain characteristics

Three components of pain were assessed: location (neck or low back), intensity (using an 11-point scale; 0 = Nothing, 10 = Intense pain) and disability related to pain (by a five-point scale extracted from the Spanish version of SF-36 questionnaire; 5 = extremely, 4 = quite a bit, 3 = moderately, 2 = slightly, 1 = not at all) [15]. Disability related to pain was coded into two groups (high/low disability) as shown in Table 2 and Table 3. Responses of 5 and 4 were considered as high disability. This cut point was decided before examination of data.

**Table 3:** Odds ratio (95%CI) of predictive factors of adherence to home exercise components of frequency per week and duration per session.

Predictive factors	Adherence to frequency per week		Adherence to duration per session	
	Univariate	Multivariate	Univariate	Multivariate
	OR (95%CI)	OR (95%CI)	OR (95%CI)	OR (95%CI)
	n = 184	n = 168	n = 158	n = 146
CHARACTERISTICS OF PATIENT				
Gender Female	0.5 (0.2-1.2)		0.6 (0.2-1.6)	
Age				
18-39	1.0		1.0	
40-59	0.8 (0.4-1.6)		0.7 (0.3-1.6)	
>59	1.1 (0.5-2.5)		0.6 (0.2-1.5)	
Education level				
Without studies	1.0		1.0	
Primary/Secondary/university	0.9 (0.4-2.1)		0.3 (0.1-1.2)	
Work participation	0.9 (0.5-1.7)		0.8 (0.4-1.6)	
Sick leave	0.8 (0.4-1.8)		0.7 (0.3-1.9)	
Use of physiotherapist in previous episodes of pain	2.0 (1.1-3.7)*		0.9 (0.4-1.8)	
Participation in home program in previous episodes				
No participation	1.0		1.0	-
Participation and low adherence	0.8 (0.4-1.5)		0.5 (0.2-0.9)*	0.3 (0.1-0.9)*
Participation and adherence	2.9 (1.1-7.9)*		1.1 (0.4-3.2)	0.5 (0.1-2.2)

CHARACTERISTICS OF PAIN				
Low back pain	0.9 (0.4-1.7)		1.3 (0.6-2.9)	
Pain intensity	1.0 (0.8-1.2)		0.9 (0.7-1.1)	
High level of disability	1.2 (0.6-2.2)		1.0 (0.5-2.1)	

CHARACTERISTICS OF HOME EXERCISE PROGRAM				
Strength exercises	0.9 (0.5-1.7)		1.1 (0.5-2.3)	
Stretching exercises	1.4 (0.6-3.5)		1.6 (0.6-4.4)	
Total number of exercises				
1-3	1.0		1.0	
4-6	0.7 (0.3-1.4)	0.6 (0.2-1.8)	0.8 (0.4-1.9)	
>6	0.3 (0.1-0.7)*	0.2 (0.1-0.9)*	0.6 (0.2-1.8)	
Days per week				
1-3	1.0		1.0	
4-6	1.5 (0.7-3.6)		2.2 (0.8-6.2)	
>6	2.0 (0.9-4.5)		1.2 (0.5-2.8)	
Duration/session				
<10 minutes	1.0		1.0	
> 10-15 minutes	0.4 (0.2-1.1)		0.6 (0.2-1.9)	

ENVIRONMENT and SELF-EFFICACY				
Have enough time	2.8 (1.5-5.2)*		1.4 (0.7-2.8)	
Exercises fit daily routine	7.4 (4.4-26)*	12.6 (0.8-19)	4.5 (1.4-14)*	
Positive emotional support	3.5 (1.5-8.1)*		2.8 (1.1-7.4)*	
Self-efficacy	1.7 (1.4-2.1)*	1.5 (1.1-2.0)*	1.4 (1.2-1.7)*	1.4 (1.1-1.8)*

PHYSIOTHERAPISTS' BEHAVIOURS IN CLINICAL ENCOUNTERS				
***General information***				
Clarifying doubts and questions from patients	2.5 (1.3-4.7)*	4.1 (1.4-12)*	1.6 (0.8-3.4)	
Giving information about illness	0.9 (0.5-1.7)		0.9 (0.4-1.7)	
Justifying usefulness of advises	1.1 (0.5-1.7)		0.7 (0.3-1.8)	
***Instructions and follow-up of exercises***				
Giving written instructions	0.8 (0.4-1.4)		1.1 (0.6-2.4)	
Supervising exercises in health care centre	1.6 (0.8-3.0)		2.7 (1.3-5.8)*	3.3 (1.3-8.6)*
Asking about adherence at home	1.5 (0.8-2.8)		1.4 (0.7-2.9)	
***Patient's assessment***				
Satisfaction	1.2 (1.1-1.4)*		1.1 (0.9-1.4)	

ADHERENCE				
Good adherence to frequency per week	---		8.0 (3.7-17)*	4.1 (1.6-10)*
Good adherence to duration per session	8.0 (3.7-17)*	4.7 (1.6-14)*	---	

* p < 0.05

#### Characteristics of HEP

Because participants were given different HEP, each physiotherapist recorded type of exercise (strength/stretching), total number of exercises per session, frequency per week (days) and estimated duration for each session of exercises.

#### Environmental factors and self-efficacy

Perceived barriers in exercising and social support were assessed as environmental factors. Perceived barriers were measured with an adaptation of the barriers subscale of Sluijs et al [5]. Social support was measured by assessing of emotional support from family or proxy. Both perceived barriers and emotional support were measured on a five-point scale (5 = always, 4 = very often, 3 = sometimes, 2 = rarely, 1 = never) and categorized on two categories with responses of 5 and 4 as coded as low perception of barrier and positive emotional support. We adapted the Spanish version of revised self-efficacy for exercise scale validated in Spain [16] to create an unidimensional self-efficacy scale by asking participants how confident they were that they could participate in HEP. Measurement was on a continuous scale ranging from 0 (minimum) to 10 (maximum).

#### Professionals' behaviors during clinical encounters and patient's satisfaction

We measured behaviors of the physiotherapist in the areas of giving general information, instructions and follow-up for exercises. Information provided was measured with three items (clarifying doubts of patient, giving information about illness, justifying advise) using two response categories (yes/no). These items were identified as relevant based on scientific literature and clinical practice guidelines pertaining to neck and low back pain care [1,17,18]. Instructions and follow-up for exercises were identified from a patient questionnaire with face validity [18]. Patient's overall satisfaction was measured using a continuous scale ranging from 0 (minimum) to 10 (maximum).

### Outcomes measures

#### Adherence

Adherence has been defined as the extent to which a person's behavior coincides with professional advice [19]. In this study adherence has been measured as compliance to each prescribed component (frequency per week and duration per session) of a patient's specific HEP. Patients who received specific advice regarding at least one of these HEP components were asked about their adherence one month following completion of physiotherapy. Adherence was measured using a frequency-based response scale (never, seldom, often, almost always, and always) adapted from the adherence scale of Sluijs et al [5] for both frequency and duration components of the HEP. Adherence is treated as a dichotomous variable (adherent or not adherent), with patients reporting that the complied "always" or "almost always" considered adherent. To avoid desirability bias, questions were phrased indirectly according to recommendations from Sackett and Haynes [19].

### Data analysis

Respondents and non-respondents to postal questionnaire were compared by baseline information using chi-square test (for gender, age, education level, working participation, sick leave, location of pain and pain-disability) and Student t-test (for pain intensity). To study statistical significance we also calculated 95% confidence interval (CI) of the difference in proportions and means between them (table 2).

Descriptive statistics using proportions and 95% CIs were calculated for adherence.

To study statistical significance of the difference in proportions between subgroups we used a chi square test.

Univariate and multivariate logistic regression analyses using two-level dependent variables of adherence, one for the frequency component and another one for the duration component, were used to assess the possible predictive factors associated with adherence to these HEP components. In the univariate analysis, associations were tested for a significant relationship (p < 0.05) with both frequency and duration adherence. In the multivariate analysis, the factors with a significant univariate contribution (p < 0.05) were combined in the total model. Using recommendation of 10 events (adherent subjects) per variable [20], considering an estimated 50% of adherence [5-10], and a maximum of nine factors with significant contribution in univariate analysis, we included 180 subjects in the study. The final models were produced by backwards elimination of independent variables with dropping out an independent variable using the likelihood ratio test at a significance level of p = 0.05. Goodness-of-fit and regression diagnostics for the reduced model were assessed using the methods described by Hosmer and Lemeshow [21]. In summary, if an independent variable with a p-value exceeding 0.05 improved model fit it was retained in the final model.

## Results

### Participants

Over a six months period we identified 317 subjects, but 50 were excluded (16 were told to stop their HEP and 34 were unable to attend all session). Thus, we had 267 eligible subjects: 104 and 163 subjects with chronic non-specific neck or low back pain, respectively. Of them, 250 (93.6%) participated in baseline interview and 184 (68.9%) returned questionnaire after treatment. Non-respondents did not differ significantly from respondents when compared by gender, education level, working participation, sick leave, location of pain, pain-disability and pain intensity. However, the proportion of patients above 59 years is higher among respondents (Table 2).

### Physiotherapy counselling

All participants received recommendations to do a home exercise program (HEP). Most of the time, the HEP included stretching and strength exercises, as well exercise components, such as frequency per week and duration per session. While all respondent patients received information about frequency only 86% of them received recommendations for duration. These participants who did not receive recommendations were not included in the analysis of factors for the duration component.

### Adherence rates

The proportion of subjects reporting adherence to frequency and duration of the HEP during the first month have slight differences in our sample, but difference in proportions was not significant (p > 0. 05). Approximately, there are 10% more subjects with adherence to component of duration per session (70.1%; CI = 63.0 to 77.2) than frequency per week (60.7%; CI = 53.7 to 67.7). Thus, patients had similar difficulty fulfilling the weekly exercise sessions of the HEP than the duration of each session.

### Predictive factors

In the univariate analysis ten factors were associated with frequency adherence (use of physiotherapist in previous episodes, participation and adherence to previous HEP, being given more than 6 exercises, environment factors and self-efficacy, clarifying doubts, satisfaction and good adherence to duration component) (Table 3). There were less univariate associations for duration adherence (participation and low adherence in another HEP, exercises fit daily routine, emotional support, self-efficacy, supervising exercises in health care centers, and adherence to component of frequency per week) (Table 3).

Table 3 presents the results of final multivariate models for both exercise components, frequency per week and duration per session. Some physiotherapist' behaviors are specific predictive factors of each exercise component. While patients who receive clarifying of doubts from physiotherapist are four times more likely to have higher levels of frequency adherence (odds ratio [OR] = 4.1; CI = 1.4-12; p < 0.05), those patients who received frequent supervision of their exercises during physiotherapy have higher levels of duration adherence (OR = 3.3; CI = 1.3-8.5; p < 0.05).

Total number of exercises per session in the HEP is a specific predictive factor for frequency adherence. Subjects who received an HEP including more than 6 exercises have lower odds of frequency adherence (OR = 0.2; CI = 0.1-0.9; p < 0.05) than those with three or less exercises.

Self-efficacy was the only common predictive factor to both frequency (OR = 1.5; CI = 1.1-2.0; p < 0.05) and duration adherence (OR = 1.4; CI = 1.1-1.8; p < 0.05). Additionally, good adherence to duration component was predictive factor to the frequency component (OR = 4.7; CI = 1.6-14; p < 0.05), and viceversa. On the other hand, while participation in previous home exercise programs is associated with the duration but not frequency adherence, total number of exercises is associated with frequency but not duration adherence.

## Discussion

This study extends several important aspects of previous knowledge about exercise adherence to HEPs and its predictive factors. Our study did not demonstrate different rates of adherence to both HEP components, frequency per week and duration per session. However, other authors have also found variability among exercise components [14].

According to our expectation, this study revealed that the predictive factors of frequency adherence were different from those associated with duration adherence. The only shared predictors were self-efficacy and participation in home exercises during previous episodes of pain. The association between self-efficacy and adherence is consistent with previous research with aerobic exercise in general population [14] and older adults [13].

The importance of barriers is consistent with the results of previous research [5,8,12,22]. Barriers related to fitting the HEP into a daily routine was included in multivariate model to explain frequency adherence, but was not significant for duration. If this finding is true, it suggests that barriers influence frequency adherence but not duration. It is reasonable to expect that barriers influence initiation of a HEP session (and therefore frequency adherence), but once a HEP session is initiated barriers are no longer important and duration adherence can be fulfilled. Just as barriers differ for different populations [23], barriers may differ for different components of HEP. For example, it is possible that physical symptoms interfere more with duration adherence that with other components of the HEP. Further research is needed to confirm these findings more fully examine differences in specific barriers to HEP components.

It is important to note that none of the patient characteristics are significant determinants of adherence in this sample. These factors have been the focus of numerous investigations of adherence in several chronic diseases. However, age, sex and education have not been definitely associated with adherence [24]. While it is possible that this may be influenced by response bias, the similarities in these factors between respondents and non-respondents make this possibility nominal for most factors. The only factor that may be impacted by response bias is that of age. Since there was greater response from the >59 years age group, we cannot rule out age >59 years as a predictor of either component of HEP adherence.

The influence of adherence to HEP on long-term adherence has been demonstrated [25]. However, our findings add that previous adherence differentially influences current frequency and duration adherence. Previous adherence predicted duration adherence, but did not predict frequency adherence.

Variables related to how physiotherapists interact and communicate with their patients are key determinants of home exercises adherence. In fact, one of the major barriers to adherence described in the literature is lack of information to the patient. However, lack of information alone is not enough for creating or maintaining good adherence habits. For example, the "Information, Motivation and Behavior (IMB) model" asserts that information is a prerequisite for changing behavior, but in itself is usually insufficient to achieve this change if the patient is not motivated to perform the behavior [26,27]. In this study, it is logical to surmise that the most motivated patients are those that ask questions during clinical encounters. Our results supports the IMB model: providing patients' required information has a decisive influence on performing home exercises to recommended frequency per week (Table 3, item "Clarifying doubts and questions from patient" increases odds of adherence to frequency multivariate OR (95% CI) = 4.1 (1.4-12)).

Intervention characteristics also have an important influence on adherence. In this sense it could be hypothesised that the greater the number of exercises prescribed for the HEP, the greater the probability that subjects do not complete them. This phenomenon is similar to those reported in other recent works about adherence in HEP and in medications for chronic conditions [28,29].

As might have been expected, there was a significant relationship between frequency and duration adherence (Table 3, item "Good adherence to frequency per week" increases odds of adherence to duration univariate OR (95% CI) = 8.0 (3.7-17) and the same for item "Good adherence to duration per session"), suggesting that when patients meet frequency recommendations it is more probable that they also regularly meet the duration recommendations.

This study has limitations. First, because variables related with physiotherapists' behaviors were measured together with adherence measures, the direction of causality cannot be determined. Thus, this is a study of association and is not a study of cause and effect. However, it should be noted that the physiotherapy adherence literature does show a causal connection of interactions between physiotherapists and patients causing an increase in adherence in home exercise programs [11].

Our analysis used the completed data in the respondent sample, which may bias the findings toward increased adherence. The more conservative intent to treat analysis (include non respondents as non adherent) was not chosen as too many assumptions would influence the analysis with imputed "non-adherence" for the non-respondents.

Nevertheless, the reader should realize that we cannot rule out the possibility that there was a predictive influence of age - which was different between the respondents and non respondents.

Finally, the statistical models presented herein have been utilized to test hypotheses regarding relationships between a variety of factors with either frequency or duration adherence to HEP. These models should not, at this time, be utilized in an attempt to predict odds of adherence in clinical populations based on these factors. Such predictions will first require additional data to confirm relationships and propose predication equations; and then would require verification in an independent sample.

## Conclusion

We have shown in a clinic-based study that adherence to HEP components appear to have distinct predictive factors and that previous findings in the general population regarding predictors of exercise components apply partially to HEPs with a clinical population. Our data indicates the possibility, in subjects with chronic non-specific neck or low back pain, that experience with previous home exercise programs, perceived barriers and self-efficacy to overcome them, number of exercises included into home program and quality of clinical encounter are associated with higher levels of adherence to exercise components of frequency per week or duration per session. It is encouraging that many of physiotherapist behaviours associated with adherence to exercise components in this study can be enhanced and the effects of changing behaviour could be formally tested in a randomised controlled trial.

Further research is necessary to examine different predictive factors of other HEP components, such as intensity or months of exercise in a year, and types of exercises. This study focused on different components of exercise but did not address different types of exercise. For example, perhaps different variables explain strength as compared with stretching exercise. Research that examines other specific exercise components and types of exercises will aid in the design of efficacious rehabilitation interventions.

## Competing interests

The authors declare that they have no competing interests.

## Authors' contributions

FMM, PER, JJGC and JMH participated in the design of the study and performed the analysis. FMM, PER and JMH secured funding for the project and participated in its coordination. PER, FJJS and SMC contributed to management of data. All authors helped to draft the manuscript, and read and approved the final manuscript.

## Pre-publication history

The pre-publication history for this paper can be accessed here:

http://www.biomedcentral.com/1471-2474/10/155/prepub
